# Strategies to Improve the Outcome of Cryoballoon Ablation in the Treatment of Atrial Fibrillation

**DOI:** 10.1155/2020/6720481

**Published:** 2020-04-06

**Authors:** Ying Huang, Yuehan Wang, Chenyu Song

**Affiliations:** ^1^Department of Cardiology, The First Affiliated Hospital of Anhui Medical University, Hefei 230022, China; ^2^Department of Electrocardiographic and Cardiac Function, The First Affiliated Hospital of Anhui Medical University, Hefei 230022, China; ^3^The First Academy of Clinical Medical, Anhui Medical University, Hefei 230032, China

## Abstract

Atrial fibrillation (AF) is a common arrhythmia contributing to severe outcomes, including cardiac dysfunction and stroke, and it has drawn great attention around the world. Drug therapies have been available for many years to terminate AF and control heart rate. However, the results from clinical studies on drug therapies have been discouraging. Mounting evidence indicates that radiofrequency catheter ablation (RFCA) is a safe and effective method to maintain sinus rhythm, especially in patients who are drug intolerant or for whom the drugs are ineffective, although it is a technically demanding and complex procedure. Fortunately, a novel application, cryoballoon ablation (CBA), with outstanding characteristics has been widely used. Great outcomes based on CBA have manifested its significant role in the treatment of AF. However, how to improve the safety and efficacy of CBA is a question that has not been well-answered. Would it be helpful to develop a different generation of cryoballoon? Is bonus freezing beneficial, or not? Is it better to prolong freezing time? Dose CBA combined with RFCA bring higher success rates? In this review, we comprehensively summarized useful applications for improving outcomes of CBA in AF patients.

## 1. Introduction

Atrial fibrillation (AF) is the most common arrhythmia in the whole world, and it not only contributes to clinical symptoms, such as palpitation and fatigue, but also induces severe complications, including cardiac dysfunction and stroke. Drug therapies have been available for many years to terminate the occurrence of AF and maintain sinus rhythm. However, the results of clinical studies have shown that drug therapies are minimally effective and have side effects, although some new drugs have been explored. In the past several years, plenty of studies have demonstrated that radiofrequency catheter ablation (RFCA) is more effective and safer than antiarrhythmic drug (AAD) therapy, especially in those patients who are drug intolerant or for whom AAD is ineffective [1]. Although great advances have been made with RFCA, it is technically demanding and requires expert, dexterous operator and the reoccurrence of AF because of discontinuity of circumferential lesions and related complications resulting from a complex “point-to-point” procedure have always perplexed doctors and patients.

Humans are always striving to invent and create novel instruments to make life easy and efficient, and the same applies to electrophysiologists' efforts to conquer AF. In 2007, a novel straightforward application of cryoballoon ablation (CBA), which is based on the understanding that pulmonary vein isolation (PVI) is the cornerstone of AF ablation, was for the first time applied among patients with paroxysmal AF (PaAF) [2]. Compared to the “point-to-point” ablated strategy, CBA has evidenced great advantages, such as a shorter learning curve, time savings, continuous and homogenous lesions, and fewer complications, which have resulted in it being promptly applied for the treatment of AF. In turn, many trials of it have demonstrated the feasibility, safety, and efficacy of CBA. For instance, the STOP AF trial [3], the CRYO versus RF trial [4], the FREEZE AF study [5], and the FIRE and ICE trial [6] have all demonstrated that CBA is equal to RFCA with respect to efficacy and safety in the treatment of PaAF. More importantly, with the development of different generations of cryoballoons and technological progress, CBA has also been used in patients with persistent AF (PeAF). A meta-analysis that included 3,527 patients with PaAF and PeAF showed that the recurrence rates after CBA or RFCA was similar [7]. A prospective cluster cohort study that involved 4,189 patients showed that the recurrence rate of PeAF did not differ between CBA and RFCA during middle-term follow up [8]. A single-center large-scale study also found that the free survival rate for atrial arrhythmias was 78.6% after a single procedure for PaAF and 72.3% for PeAF at a median 39 months follow-up [9]. CBA treatment also achieved a similar success rate without obvious complications compared to RFCA both in elderly AF patients (>75 years old) and patients with PeAF and longstanding PeAF [10, 11]. Collectively, CBA has become one of the main pillars and is recommended as the first-line therapy in the management of patients with AF [12, 13].

Because we are never satisfied with what we have achieved, shorter procedure times and fewer cryoapplications with better clinical outcomes are always pursued when CBA is viewed as a solid alternative in the treatment of AF. In the past few years, a large number of studies have aimed at improving the outcomes of CBA. However, how to improve the safety and efficacy of CBA is a question that has not been well-answered. Would it be helpful to develop a different generation of cryoballoon? Is bonus freezing beneficial, or not? Is it better to prolong freezing time? Does CBA combined with RFC bring higher success rates? In this review, we comprehensively summarized the useful applications for improving outcomes of CBA in AF patients.

## 2. Development of the Cryoballoon Catheter Is Helpful

The first-generation cryoballoon (CBG1) was characterized by an eccentric cooling property, which led to the formation of incomplete lesions, especially for inferior PVs, resulting in chronic PV reconduction and a high rate of AF recurrence, although acute PVI was also achieved. Thus, the second-generation cryoballoon (CBG2) with a hemispherical cooling surface that provides a large contact area was approved in 2012. Compared to CBG1, CBG2 did not increase PVI-associated complications and was a shorter procedure, requiring reduced fluoroscopy exposure and time to isolation (TTI) of PV potential. Moreover, it significantly improved the success rate of freedom from AF. In PaAF patients, success rates of 84% and 86.7% in years 1 and 2 of follow-up, respectively, were reported in the CBG2 group, compared with success rates of 66% and 68.3%, respectively, in the CBG1 group [14, 15]. In patients with PaAF and PeAF, rates of freedom from AF after using CBG1 were 64.3% and 51.3% at 1- and 2-year follow-ups, respectively, compared with 78.6% and 72.6% for CBG2 [16]. In comparison to CBG1, the more maneuverable angulations of CBG2 with an optimal freeze zone and the more homogenized cooling effect with 8 injection ports improve the success rate of durable PVI, which may be a conceivable explanation for the encouraging outcomes. However, other findings challenge these results. The prospective multicenter, multinational FREEZE Cohort Substudy found that 95% of patients in the CBG1 group and 94.8% in the CBG2 group were without atrial arrhythmias, and no significant differences between the treatments were detected during a shorter follow-up [17]. A 2-year follow-up study revealed that the rates of freedom from arrhythmia were similar across the two groups: 72.0% in both the CBG1 group and the CBG2 group in PaAF patients [18]. Hence, more details from large-scale randomized controlled trials are needed in the future to confirm the outcome of AF ablation by CBG2.

**Figure 1:**
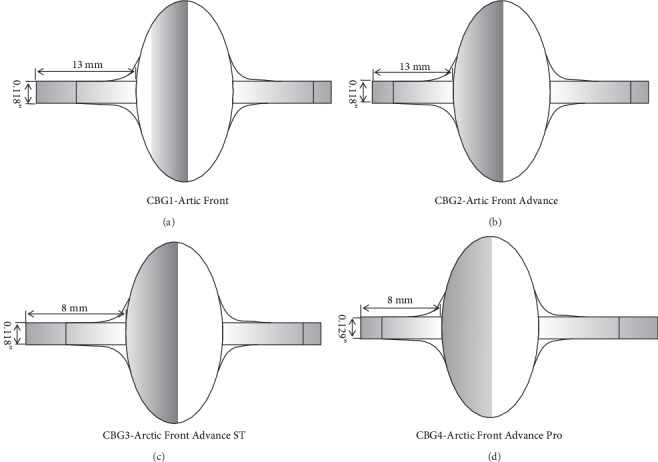
The changes among the four-generation cryoballoons. The CBG1 displayed an equatorial cooling band (shaded area) (a). Better cryoablation effect was realized due to the hemispherical cooling surface in CBG2 to CBG4 compared to CBG1 (b–d). A shortened tip contributed to better achievement of PV signals in CBG3 and CBG4 (c and d). However, the CBG4 had a larger distal tip diameter but the maximum outer diameter is the same with the previous cryoballoons (d).

To obtain stable contact in PV ostium, CBG1, and CBG2 are often positioned more distally than the PV sleeve extension. However, the long distal tip of the cryoballoon prevents the real-time visualization of PV potentials. Consequently, the novel third-generation cryoballoon (CBG3), with a 40% shorter distal tip, was developed, which increased the rate of real-time PV signal recording. To estimate the safety and efficacy of CBG3, in 2015, the first human-subjects clinical study reported that a higher rate of real-time PV potential recordings and a shorter mean freeze duration were observed while using CBG3, compared to CBG2 [19]. The multicenter study further demonstrated that CBG3 was associated with shorter left atrial dwell and procedure time, while procedure-related complications and the rate of atrial arrhythmias were similar compared to CBG2 at 10-month follow-up [20]. It is generally agreed that CBG3 greatly increased the success rate of real-time PV signal recordings, especially for the right PVs, which undoubtedly reduced procedure time. However, CBG3 may offer limited help for improving the success rate of AF ablation compared to its predecessor, based on previous findings. In 2018, the fourth-generation cryoballoon (CBG4) appeared. Compared to CBG2, CBG4 possesses a larger distal tip diameter, but the maximum outer diameter is the same (Figure 1). Use of CBG4 with the latest 20 mm spiral-mapping catheter is safe and effective, although the spiral-mapping catheter was occasionally changed to a stiff wire to acquire stable contact [21]. Recently, an interesting study evaluated four generations of CBG in the treatment of AF. The CBG4 application showed a higher rate of TTI visualization, faster procedural ablation times, and a lower rate of acute complications in comparison to the previous generations [22], while the long-term outcomes associated with CBG4 are still unclear.

## 3. Bonus Freezing Is Challenged

It has been confirmed that bonus freeze procedures enhance the lesion area and depth of PV ostia, and they induce a chronic PVI and a high rate of freedom from atrial arrhythmia. With a bonus freeze-cycle using CBG2 after PVI, almost 72% of PaAF and short PeAF patients were still free from AF at the 2-year follow-up [23]. However, this exciting and encouraging finding did not support the development of the bonus freeze protocol. Other research found that, after a signal freeze, almost 80.4%-84.6% of PaAF and PeAF patients were free from atrial arrhythmias at a midterm follow-up without increased complications [24–26]. To compare the outcomes between a signal and bonus freeze delivery, control studies were also carried out that found no significant differences in rates of success, reoccurrences, or complications. For instance, the PaAF and PeAF patients' rates of freedom from AF reached more than 80% with the nonbonus procedure and 79% with the bonus freeze method at the 1-year follow up. Moreover, the success rates were 67% for nonbonus patients and 69% for bonus patients at the 2-year follow up [27, 28]. Strikingly, a prospective multicenter randomized study on PaAF patients revealed that the total number of freeze cycles and durations were significantly shorter in the single freeze group than that in the bonus freeze group, while rates of freedom from AF were 87.3% in the bonus group and 89.1% in the nonbonus group at the 1-year follow up [29]. Taken together, no obvious benefit was found for patients receiving additional freeze cycles after the complete PVI, suggesting to some extent that bonus freezing is unnecessary and time-consuming.

## 4. Longer Time Is Futile to Recurrence

In 2013, an animal study first revealed no differences in acute efficacy or transmural lesions between 2 min and 4 min applications [30]. In clinical practice, the real freeze time is changed depending on the TTI and nadir temperature, although a 3 and 4 min freeze cycle is a currently common strategy for each PVI. For PaAF patients, a 4 min CBA has been associated with increased rates of PVI durability, particularly for left-sided PV, without increasing complications when compared to 3 min CBA, whereas the outcomes for two groups have been similar [31, 32]. In PaAF and PeAF patients, a 3 min freeze protocol is similarly safe and effective as a 4 min freeze time, and it is associated with 78.6%-85.6% of patients remaining sinus arrhythmia-free compared to 67%-87% success rates during the short- and midterm follow-ups [33, 34]. One study with a long-term follow-up of more than 3 years showed that a single 3 min freeze strategy is effective, and it can be successful in 72.8% of PaAF patients and 59.1% of PeAF patients [35]. Therefore, there are no differences in acute success, rates of complications, or rates of freedom from AF recurrences between the two groups, while the procedural and fluoroscopy times were greatly decreased with the 3 min procedure.

## 5. Combination of CBA and RF Is Incrementally more Efficacious than Either CBA or RFCA Alone

In 2010, an interesting study with 32 PeAF patients is aimed at evaluating the safety and efficacy of combining CBA and open-irrigation RFCA. The stepwise procedure first entailed PVI using CBG, followed by CFAE ablation and finally linear ablation on the roof, mitral isthmus, and septum if AF could not be terminated or there was some other atrial arrhythmia occurrence. Although this combined approach was time-consuming and complex at that time, it resulted in 86.4% of patients being AF-free without AADs at short-term follow-up, which was greatly favorable in comparison with previous reports of CBA alone for PeAF [36]. A long-term follow-up study also found similar results associated with the roofline and tricuspid isthmus ablation in which 70.3% of PeAF patients were AF-free at the 37-month follow up after PVI using CBG2 [37]. A multicenter retrospective nonrandomized study revealed that CBG2 coupled with RFCA had a 76.6% success rate at 12 months compared to 60.4% for RFCA alone in PaAF and PeAF patients [38]. In the following years, several studies further demonstrated the advantages of a combination of CBG and RFCA [39, 40]. Recently, a novel study revealed that the combination of CBA and RFCA had a significantly higher single-procedure success rate with fewer reconnected PVs and fewer reconnection sites compared to either CBA or RFCA alone. At 5 years, 57% of patients who received the combined treatment remained free of AF after a single procedure compared to 47% of CBA-alone and 19% of RFCA-alone patients [41]. Taken together, CBG coupled with RFCA can achieve safety and efficacy and have greater benefits than either RFCA or CBA alone, although the latter is supplementary if necessary.

## 6. Other Useful Devices and Methods for CBA

### 6.1. Small Balloon Diameter Is Selective

CBA application can sometimes be challenging because of the anatomical variation of PVs. Currently, 23 mm and 28 mm diameter balloons are the main styles used in CBGs. For 23 mm balloons, PVI mostly occurs in the tubular part of the ostium, while the 28 mm balloon creates a larger lesion in the left atrium [42]. The 28 mm CB is conventional for all PV, whereas 23 mm cryoballoons may be an appropriate option for patients with small PV diameters to achieve better contact and effective PVI [43, 44].

### 6.2. TEE and ICE Are Helpful

PV occlusion is commonly achieved with the help of contrast fluid injections, while it is time-consuming and limited in patients with renal dysfunction and allergic reactions. Studies have demonstrated that transesophageal echocardiography (TEE) has become available for clinical practice to provide real-time visualization of PV ostia and neighboring atrial structures [45]. However, the use of TEE requires general anesthesia, and it yields moderate to low visualization in 46% of PVs owing to the poor echograph window. Fortunately, intracardiac echocardiography (ICE) is very helpful for selecting adequate balloon size and optimal occluded position. ICE plus fluoroscopy guidance obviously has lower fluoroscopy, contrast, and procedure times compared to fluoroscopy alone during CBA procedures, but both applications have similar success rates without increasing complications [46–48]. Economic burden and the demand for special expertise are the main barriers to the wide use of ICE.

### 6.3. Pressure-Guided Is Useful

PV occlusion may be predicted by changes in the pressure curve recorded at the tip of the cryoballoon catheter. When PV is completely occluded, TEE detects blood reflux of PV, and a pulmonary artery pressure curve appears. A combination of pressure and TEE-guided cryoballoon technique is similarly effective and safe as conventional CBA. A combined approach provides real-time monitoring of the effects of catheter handling and further facilitates optimal occlusion, and its procedure and fluoroscopy times are shorter than those of RF energy, and they are also shorter than those of TEE-guided ablation only [49, 50].

## 7. Conclusion

It is well-known that CBA has become the first-line therapy for both PaAF and PeAF patients based on previous studies. Plenty of evidence seems to focus on several main points. First, CBG with a short tip is safe and can greatly increase the real-time visualization of PV potential, but it does not augment the success rate of freedom from AF. Second, a bonus freeze protocol is unnecessary if PVI is achieved by a signal freeze, and 3 min CBA is as effective as 4 min CBA based on the long-term outcome. Third, a combination of CBA and RFCA is better than either CBA or RF alone, but it increases the financial burden. Finally, the use of TEE and ICE with a pressure-guided technique is helpful for improving the ablated process but not the outcome. Of note, in clinical practice, an individual ablated strategy should always be considered for counterbalancing the risk and success.
